# Crystal structure of *Schistosoma mansoni* cathepsin D1 in complex with a nanobody reveals the conformation of the propeptide-bound state

**DOI:** 10.1107/S2059798326000422

**Published:** 2026-01-28

**Authors:** Kelly L. Parker, John D. Clarke, Xiaojiao Liu, Barbara F. Gomes, Lauren E.-A. Eyssen, Nicholas Furnham, Floriano Paes Silva-Jr, Raymond J. Owens

**Affiliations:** a Rosalind Franklin Institute, Rutherford Appleton Laboratory, Harwell Campus, Didcot, United Kingdom; b Department of Infection Biology, London School of Hygiene And Tropical Medicine, London, United Kingdom; c Lonza Biologics, 228 Bath Road, Slough, United Kingdom; d LaBECFar – Laboratory of Experimental and Computational Biochemistry of Drugs Oswaldo Cruz Foundation (FIOCRUZ) Rio de Janeiro Brazil; e Division of Structural Biology, The Centre for Human Genetics University of Oxford Oxford United Kingdom; McGill University, Canada

**Keywords:** *Schistosoma mansoni*, cathepsin D, nanobodies, X-ray crystallography

## Abstract

*Schistosoma mansoni* cathepsin D1 (*Sm*CD1) has been shown to be an essential enzyme for helminth metabolism due to its role in haemoglobin degradation: a key amino-acid source for the developing parasite. Therefore, the enzyme is a potential target for the development of antischistosomal inhibitors. *Sm*CD1 has significant sequence identity to cathepsin D-like proteases found in other schistosome species and homology to mammalian aspartic proteases. Here, we report the first crystal structures of a helminth cathepsin D, *Sm*CD1, and have identified a single-domain antibody (nanobody) that specifically binds to *Sm*CD1 with nanomolar affinity but does not recognize human cathepsin D. We have mapped the epitope of the nanobody by determining the crystal structure of the enzyme–nanobody complex, revealing the conformation of *Sm*CD1 in the propeptide-bound state.

## Abstract 2

Structural characterization of *S. mansoni* cathepsin D1 in complex with a nanobody reveals the conformation of the propeptide-bound state and identifies a schistosome-specific epitope.

## 1. Introduction

Schistosomiasis presents a devastating global health challenge, prevalent in sub-Saharan Africa, and is classified as a widespread neglected tropical disease (NTD) by the World Health Organization (WHO; Van Der Werf *et al.*, 2003 ▸; Steinmann *et al.*, 2006 ▸; World Health Organization, 2021 ▸). Of the three major species infecting humans, *Schistosoma mansoni* is causative of acute intestinal and hepatic schistosomiasis (Han *et al.*, 2009 ▸). Despite its significant impacts, existing treatments for schistosomiasis are heavily reliant on mass administration of praziquental (PZQ), which shows negligible effects against juvenile parasitic stages and introduces challenges for drug resistance (Humphries *et al.*, 2012 ▸; Xiao *et al.*, 2018 ▸; Aboagye & Addison, 2022 ▸). Similarly, current antibody diagnostic tests exhibit variable accuracy for low-intensity infections, displaying inconsistent sensitivity between schistosome species, and lack of correlation to clinical status (Valli *et al.*, 1997 ▸; Ochodo *et al.*, 2015 ▸; Wilson, 2017 ▸; Ogongo *et al.*, 2022 ▸). Thus, there remains a need for further study to aid understanding and contribute to the development of accessible, sensitive tools for rapid detection and neutralization (Ajibola *et al.*, 2018 ▸; Molehin *et al.*, 2022 ▸; Chala, 2023 ▸).

Helminth aspartyl proteases have been extensively reported as playing crucial roles in parasite survival, particularly in the developing trematode (Cesari *et al.*, 1998 ▸; Liu *et al.*, 2014 ▸; Brindley *et al.*, 2001 ▸). *S. mansoni* cathepsin D1 (*Sm*CD1) is considered a potential therapeutic target because of the pivotal role that the enzyme plays in host haemoglobin degradation and parasite maturation, as indicated by RNAi studies (Morales *et al.*, 2008 ▸). *Sm*CD1 is highly conserved across species, including >82% shared identity with orthologues from *S. japonicum* and *S. bovis*, and retains significant sequence similarity to human cathepsin D (hCD) and other mammalian and invertebrate homologues (Morales *et al.*, 2004 ▸; Silva *et al.*, 2011 ▸; Kang *et al.*, 2019 ▸). *Sm*CD1 is known to be expressed within the gut of the developing parasite, where the acidic environment is crucial for enzyme activation (Timms & Bueding, 1959 ▸; Koehler *et al.*, 2007 ▸; Figueiredo *et al.*, 2015 ▸). In common with other aspartyl proteases, the propeptide of *Sm*CD1 is presumed to occupy the active site of the enzyme and is cleaved upon activation at acidic pH, permitting the access of substrates to the catalytic aspartyl residues. There is evidence that *Sm*CD1 exists as a dimer, which shifts to a monomeric state upon activation at acidic pH (Araujo-Montoya *et al.*, 2020 ▸). The functional implications of this reversible interaction have yet to be determined, and the dimerization interface remains to be definitively identified.

Despite the potential of *Sm*CD1 as a therapeutic target, structural characterization of the enzyme is lacking and there are limited studies utilizing *Sm*CD1 to formulate novel agents (Dougall *et al.*, 2014 ▸; Gomes *et al.*, 2023 ▸; Balogun *et al.*, 2024 ▸). Here, we report crystal structures of *Sm*CD1 alone and in complex with a high-affinity nanobody, which stabilizes the propeptide of the enzyme, providing insight into the conformation of the *Sm*CD1 zymogen.

## 2. Materials and methods

### 2.1. Production of recombinant *Sm*CD1 and human homologues

The genes encoding *Sm*CD1_15–385_ (UniProt G4VEV6), human cathepsin D_211–412_ (UniProt P07339) and human cathepsin E_20–398_ (UniProt P14091) were inserted into the vector pOPINTTGneo by In-Fusion cloning (Berrow *et al.*, 2007 ▸), which adds an N-terminal mu-phosphatase signal sequence and a C-terminal 6×His tag (Nettleship *et al.*, 2009 ▸). Recombinant cathepsins were expressed in Expi293 cells (Le Bas *et al.*, 2022 ▸) and purified by tandem affinity size-exclusion chromatography with HisTrapFF (Cytiva) and HiLoad 16/600 Superdex 200 columns (Cytiva) on an ÄKTAxpress HPLC system (Nettleship *et al.*, 2009 ▸). Eluted fractions were analysed by reducing SDS–PAGE, pooled and mass-validated by mass spectrometry. For crystallization, *Sm*CD1 was expressed in the presence of kifunensine (1 µg per millilitre of culture) to inhibit complex glycosylation (Chang *et al.*, 2007 ▸).

The activity of purified *Sm*CD1 was assayed as described previously (Araujo-Montoya *et al.*, 2020 ▸) using the FRET substrate [7-methoxycoumarin-4-acetyl-GKPILFFRLK(DNP)-d-R-amide]. Briefly, enzyme (1 µ*M*–100 n*M* in 20 m*M* sodium acetate, 150 m*M* NaCl buffer at pH 3.0–7.5) was incubated with the substrate (1 µ*M*) in a final reaction volume of 100 µl. Immediately after adding substrate, relative fluorescence units (RFU) at an excitation of 320 nm and emission of 420 nm were measured with a CLARIOStarPlus Plate Reader (BMG Labtech), recording every 30 s for 2 h. For inhibition studies, 100 n*MSm*CD1 was incubated with a twofold excess of Nb10C9. RFU data were analysed in *GraphPad Prism* (v.10.3.1) and linear regression was used to calculate the slope of fluorescence over time.

### 2.2. Generation of nanobodies to *Sm*CD1

Nanobodies to *Sm*CD1 were generated as described by Eyssen *et al.* (2024 ▸), adapted for solid-phase panning as outlined by Zimmermann *et al.* (2020 ▸). One llama was immunized in accordance with CEDAR policies and adhering to Animal Research Reporting of In Vivo Experiments (ARRIVE) principles. Blood sampling was conducted following the UK Government Animal (Scientific Procedures) Act 1986 under UK Home Office project licence PA1FB163A. For immunization, 200 µl of 1 mg ml^−1^ purified *Sm*CD1 was administered on day 0, followed by two boosts of 200 µg antigen per immunization on days 28 and 56. Ten days following the final boost, a 170 ml blood draw was taken. A seroconversion ELISA confirmed the camelid immune response to *Sm*CD1 (Supplementary Section S1.1). Peripheral blood mononuclear cells (PBMCs) were isolated from blood samples by density-gradient centrifugation. RNA was extracted from PBMCs and used as a template for rtPCR to amplify sequences corresponding to the variable domain of heavy-chain-only antibodies (VHH or nanobodies). Following PCR, nanobodies were cloned into the phagemid-display vector pADL23c to prepare a phagemid-display library. Phage-displaying nanobodies specific for *Sm*CD1 were enriched by two rounds of bio-panning with 50 and 10 n*MSm*CD1, respectively, confirmed by polyclonal ELISA (Supplementary Section S1.1). 93 individual phage clones were picked from each round of panning; nanobody-displaying phages were recovered by infection with M13K07 helper phage and tested for binding to *Sm*CD1 by anti-M13 ELISA (Supplementary Section S1.1). Positive phage binders were sequenced and analysed for CDR3 sequence identity using *IMGT*/*V-QUEST* (Giudicelli *et al.*, 2011 ▸). Nanobodies were subcloned into the pOPINVHH_his vector (Eyssen *et al.*, 2024 ▸) by In-Fusion cloning (Berrow *et al.*, 2007 ▸) for the production of proteins for biophysical and structural studies.

### 2.3. Production of nanobodies and complex with *Sm*CD1

Nanobodies were first expressed on a small scale using WK6 cells in 4 ml Terrific Broth medium supplemented with 100 µg ml^−1^ ampicillin. Cell pellets were resuspended in 300 µl 1 mg ml^−1^ polymyxin B sulfate (Sigma–Aldrich) to isolate the periplasm. Nanobodies were purified using Ni–NTA spin columns (Qiagen) and desalted with Zeba desalting columns (Thermo Scientific). Purified nanobodies were analysed on SDS–PAGE and binding was evaluated by titration ELISA (Section 2.4).

To obtain a higher yield for further characterization, nanobodies were expressed on a large scale using WK6 cells in 1 l Terrific Broth medium supplemented with 100 µg ml^−1^ ampicillin, 0.1%(*v*/*v*) glucose and 2 m*M* MgCl_2_. The periplasm was isolated by osmotic shock with TES buffer, and nanobodies were purified by tandem affinity size-exclusion chromatography with HisTrapFF (Cytiva) and HiLoad 16/600 Superdex 75 columns (Cytiva) on an ÄKTAxpress HPLC system as per established protocols (Nettleship *et al.*, 2009 ▸; Le Bas *et al.*, 2022 ▸). Eluted fractions were analysed by reducing SDS–PAGE, pooled and validated for size by mass spectrometry.

For crystallization trials, *Sm*CD1 was mixed with a 1.2 molar excess of Nb10C9 for 3 h at 4°C and agitated at 25 rev min^−1^. Following this, Endo F1 was added at 0.05 molar equivalents relative to the total protein and incubated at 4°C overnight to remove high-mannose glycoforms. The sample containing *Sm*CD1, Nb10C9 and Endo F1 was first manually passed over a GSTrap glutathione *S*-transferase affinity column (Cytiva) equilibrated with 20 m*M* reduced glutathione diluted in SEC buffer (20 m*M* Tris, 300 m*M* NaCl pH 7.5) to remove Endo F1 and cleaved oligosaccharides. The *Sm*CD1–Nb10C9 complex was co-eluted using a Superdex 200 10/300 GL Increase column (Cytiva) equilibrated in 20 m*M* Tris, 300 m*M* NaCl pH 7.5 on an ÄKTApure HPLC (Le Bas *et al.*, 2022 ▸). Fractions were assessed for the presence of the complex by reducing SDS–PAGE and those of interest were spin-concentrated with a Vivaspin 20 10 000 MWCO column (Sartorius). Co-purified complex was stored at 4°C and used for crystallization screening on the same day as purification.

### 2.4. ELISA and biolayer interferometry

Small-scale purified nanobodies were analysed for binding to *Sm*CD1 by α-VHH titration ELISA as described in Eyssen *et al.* (2024 ▸), using wells coated overnight at 4°C with 50 n*MSm*CD1. Binding was assessed using 25 µg ml^−1^ as the highest concentration of nanobody with a twofold dilution series. Following successful large-scale purification, a further α-VHH titration ELISA was performed with Nb10C9, starting with a concentration of 20 n*M*. To assess potential cross-reactivity to human cathepsin D (hCD) and human cathepsin E (hCE), ELISA wells were coated overnight at 4°C with 50 n*M* antigen diluted in PBS. Nb10C9 was titrated from 1 µ*M* in a series of tenfold dilutions. For evaluation of nanobody pH-sensitivity, ELISAs were adapted as per Supplementary Section S1.2.

For all ELISAs, wells were washed thrice between incubations with 300 µl PBST. 0.1 µg ml^−1^ Monorab Rabbit Anti-Camelid VHH-HRP monoclonal antibody [GenScript, catalogue No. A01861; diluted in 0.1%(*w*/*v*) BSA–PBS] was added to detect bound nanobody. ABTS peroxidase substrate (LGC SeraCare) was added for antibody capture and incubated in the dark for 15 min. Absorbance was measured as microplate endpoint at 405 nm using a CLARIOStarPlus plate reader (BMG Labtech). ELISA data were analysed using *GraphPad Prism* (v.10.3.1).

Biolayer interferometry (BLI) was used to estimate a dissociation constant (*K*_d_) as a measure of nanobody affinity. All experiments were designed using *Octet BLI Discovery Software* (v.13.0) and were conducted on an Octet R8 system (Sartorius). Assays were performed at 30°C using 0.1%(*w*/*v*) BSA–PBST as the assay buffer. Biosensors were first washed in buffer-only wells for 600 s to generate a baseline. *Sm*CD1 was biotinylated using a One-Step Antibody Biotinylation Kit (Miltenyi Biotec) and free biotin was removed using Zeba Biotin Removal Columns (Thermo Scientific). 50 n*M* biotinylated *Sm*CD1 was immobilized on streptavidin biosensors during a 300 s loading step. After a 60 s wash and 30 s re-equilibration into buffer, sensors were incubated with wells containing nanobody titrated from 200 n*M* in a series of twofold dilutions. Association was measured for 600 s and dissociation was measured in buffer-only wells for a further 600 s. Data points were collected every 0.2 s. Kinetic characterization was completed in *Octet Analysis Studio* (v.13.0). Graphs were generated in *GraphPad Prism*, with data represented as nanometre shift plotted against time. Replicate BLI measurements were combined.

### 2.5. Crystallization and structure determination

For crystallization of the *Sm*CD1 enzyme alone, droplets consisting of 100 nl protein solution at 20 mg ml^−1^ and 100 nl reservoir solution were dispensed into MiTeGen *In Situ*(2-drop) plates using a Mosquito liquid-handling robot (TTP Labtech). Crystals were obtained using sitting-droplet vapour diffusion at 20°C (293 K) in the presence of 0.1 *M* bis-Tris propane pH 8.5, 0.02 *M* sodium/potassium phosphate, 20%(*v*/*v*)PEG 3350. For the *Sm*CD1–Nb10C9 complex, crystal screening was also carried out by sitting-droplet vapour diffusion. Droplets consisting of 50 nl protein complex at 10 mg ml^−1^ and 100 nl reservoir solution were dispensed as described previously. Crystals were obtained at 20°C (293 K) in the presence of 0.1 *M* PCTP pH 6.00, 0.2 *M* CaCl_2_, 20%(*v*/*v*) PEG 3350. X-ray diffraction (XRD) data for all structures were collected *in situ* to 1.95 Å resolution at 293 K using a Dectris EIGER2 X 4M detector at the VMXi instrument, Diamond Light Source (Sanchez-Weatherby *et al.*, 2019 ▸; Mikolajek *et al.*, 2023 ▸).

The data set for *Sm*CD1 alone was produced from a single crystal using *xia*2/*DIALS* (Winter, 2010 ▸; Winter *et al.*, 2022 ▸; Gildea *et al.*, 2022 ▸), as implemented in the Diamond Light Source *SynchWeb* interface ISPyB (2025-R.4). The data set was extended to 2.20 Å resolution, yielding a corrected *R* factor of 0.292 overall and 0.094 for the inner shell. All shells had 100% completeness, and CC_1/2_ for the outer shell was 0.311 (Table 1 ▸).

The final data set for *Sm*CD1–Nb10C9 was produced by multiplexing 18 data sets using *xia*2/*DIALS* (Winter, 2010 ▸; Winter *et al.*, 2022 ▸; Gildea *et al.*, 2022 ▸), again implemented in the Diamond Light Source *SynchWeb* interface ISPyB (2025-R.4). The multiplexed enzyme data set was extended to 2.59 Å resolution, yielding a corrected *R* factor of 0.671 overall and 0.165 for the inner shell. All shells had 100% completeness, and the CC_1/2_ for the outer shell was 0.262 (Table 1 ▸).

All diffraction data were phased and the atomic model was built and refined using the *Phenix* suite (Liebschner *et al.*, 2019 ▸) or the *CCP*4 suite via the *CCP*4*i*2 interface (*CCP*4 v.9.0, *CCP*4*i*2 v.1.1.0; Winn *et al.*, 2011 ▸; Agirre *et al.*, 2023 ▸) for the enzyme and the enzyme–nanobody complex, respectively.

Phasing was performed by molecular replacement using a search structure derived from the experimentally solved structure of human cathepsin D (PDB entry 1lya; Baldwin *et al.*, 1993 ▸) using *Phaser-MR* v.2.7.0 (Read, 2001 ▸). Density modification and phase improvement produced interpretable electron-density maps, which were used for automated model building using *Phenix AutoBuild*. The resulting model was manually refined through iterative cycles of model adjustment in *Coot* (Emsley *et al.*, 2010 ▸) and refinement in *phenix.refine* (Afonine *et al.*, 2012 ▸). Model quality was assessed using *MolProbity* (Chen *et al.*, 2010 ▸) and *R*-factor statistics (*R*, 0.17; *R*_free_, 0.20), and the final structure was validated against the experimental data. Coordinates and structure factors were deposited in the Protein Data Bank with code 9snt. All data were included throughout refinement, and consequently the resolution of the refined structure was 2.20 Å (Table 1 ▸).

The refined *Sm*CD1 structure and a predictive model of Nb10C9 generated with *NanoBodyBuilder*2 (Dunbar *et al.*, 2016 ▸; Abanades *et al.*, 2023 ▸) were used as initial search models with *Phaser-MR* v.2.7.0 (Read, 2001 ▸) for structural determination of the SmCD1–Nb10C9 complex. The positioned model from *Phaser-MR* was iteratively rebuilt and refined using *WinCoot* v.9.8.95 (Emsley *et al.*, 2010 ▸) and *REFMAC*5 v.5.5 (Vagin *et al.*, 2004 ▸; Murshudov *et al.*, 2011 ▸). All data were included throughout refinement, and consequently the resolution of the refined structure was 2.59 Å (Table 1 ▸).

Following structural refinement, *PDBePISA* was used for the estimation of intermolecular interactions (Krissinel & Henrick, 2007 ▸), identifying two potential binding epitopes for Nb10C9 on *Sm*CD1. Identical interactions between Nb10C9 and *Sm*CD1 were predicted using the *Protein–Ligand Interaction Profiler* (*PLIP*; Adasme *et al.*, 2021 ▸). Graphical representations were generated using *PyMOL* (v.3.0.4; Schrödinger) and *LigPlot*+ v.2.2.9 (Laskowski & Swindells, 2011 ▸).

### 2.6. Generation of Nb10C9 mutants

Knockout constructs were designed with the proposed interacting residues of the nanobody mutated to alanines. Nb10C9_KO1 was mutated at Gln1, Gln3, Tyr95, Leu122, Thr124 and Gln125. Nb10C9_KO2 was mutated at Gly26, Arg27, Thr28, Tyr32, Asn77, Tyr100 and Glu103. The gBlock templates were purchased from IDT (Integrated Technologies Inc.) and cloned into the pOPINVHH_his vector by In-Fusion cloning (Berrow *et al.*, 2007 ▸).

Mutants were expressed on a large scale using WK6 cells in 1 l Terrific Broth medium as described previously (Section 2.3). To assess binding, ELISA wells were coated with 50 n*MSm*CD1 at 4°C. Mutant nanobodies were titrated, alongside wild-type Nb10C9, from 100 µ*M* in a series of tenfold dilutions. For the detection of bound nanobody, 0.1 µg ml^−1^ anti-camelid VHH-HRP [GenScript; diluted in 0.1%(*w*/*v*) BSA–PBS] was added. ABTS peroxidase substrate (LGC SeraCare) was added for antibody capture and incubated in the dark for 15 min. Absorbance was measured as microplate endpoint at 405 nm using a CLARIOStarPlus plate reader (BMG Labtech). ELISA data were analysed using *GraphPad Prism* (v.10.3.1).

## 3. Results

### 3.1. Structure of *Sm*CD1

Recombinant *Sm*CD1, lacking the intrinsically disordered carboxy-terminal extension that appears to be unique to the *S. mansoni* enzyme (Wong *et al.*, 1997 ▸), was produced by transient expression in Expi293 cells in the presence of kifunensine. Purified *Sm*CD1 was treated with EndoF1 to remove high-mannose sugars and the protein crystallized at pH 8.5. The structure of *Sm*CD1 was determined by molecular replacement using a search structure derived from the experimentally solved structure of human cathepsin D (PDB entry 1lya) to a resolution of 2.20 Å (Table 1 ▸). A single copy of *Sm*CD1 was placed within the asymmetric unit, consisting of 14 α-helices and 25 β-sheets which form two domains stabilized by three disulfide bonds and typical of aspartyl proteases, with each domain contributing an aspartate residue to the active site (Asp84 and Asp270). Connective density was observed for most of the propeptide sequence (Val15–Leu45) which forms the sixth β-strand in the inter-domain β-sheet. However, the electron-density map was insufficient to distinguish the remainder of the propeptide-including region, where it is cleaved on enzyme activation (Ser46–Pro53; Supplementary Fig. S1). The S2 subsite residues (Met336 and Gly337) of the protease (Silva *et al.*, 2002 ▸) are also not resolved in the crystal structure. The catalytic aspartic acids and active-site flap of *Sm*CD1 were positioned in equivalent locations as in human cathepsin D (PDB entry 1lyb; Baldwin *et al.*, 1993 ▸), exhibiting an average r.m.s.d. of 0.72 Å. In addition, homology is observed across the cathepsin gene family, with human cathepsin E sharing 46.7% identity with *Sm*CD1 (63.6% similarity; Supplementary Fig. S2). Notably, human cathepsin E (PDB entry 1tzs; Ostermann *et al.*, 2004 ▸) also shows a markedly similar overall structure to *Sm*CD1, with an average r.m.s.d. of 0.87 Å over all residues, with a short stretch of the propeptide also present in the structure.

**Figure 1:**
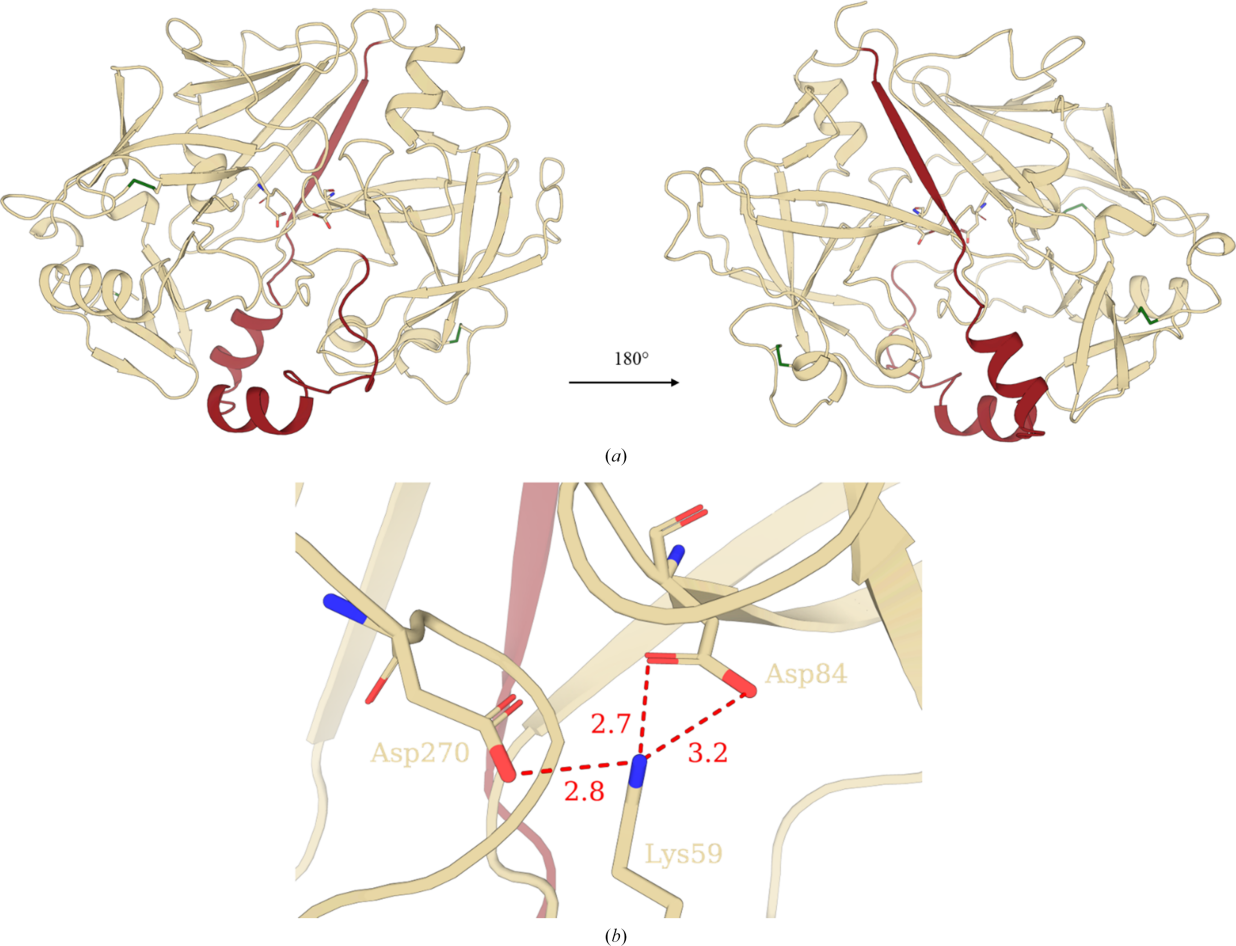
Structure of *Sm*CD1. (*a*) Overall structure of the *Sm*CD1 backbone with the propeptide sequence shown in maroon, disulfide bridges in green and catalytic aspartates shown in stick representation coloured by element. (*b*) Salt-bridge interactions between Asp84, Asp270 and Lys59.

Overall, *Sm*CD1 was observed in an inactive conformation, as expected from the basic pH of the crystallization conditions, with the propeptide occluding the active-site cleft and the N-terminal strand (residues Gln54–Tyr67) inserted into the catalytic site, with Lys59 forming salt bridges with the two active-site aspartates (Fig. 1 ▸).

### 3.2. Identification and characterization of a nanobody to *Sm*CD1, Nb10C9

**Figure 2:**
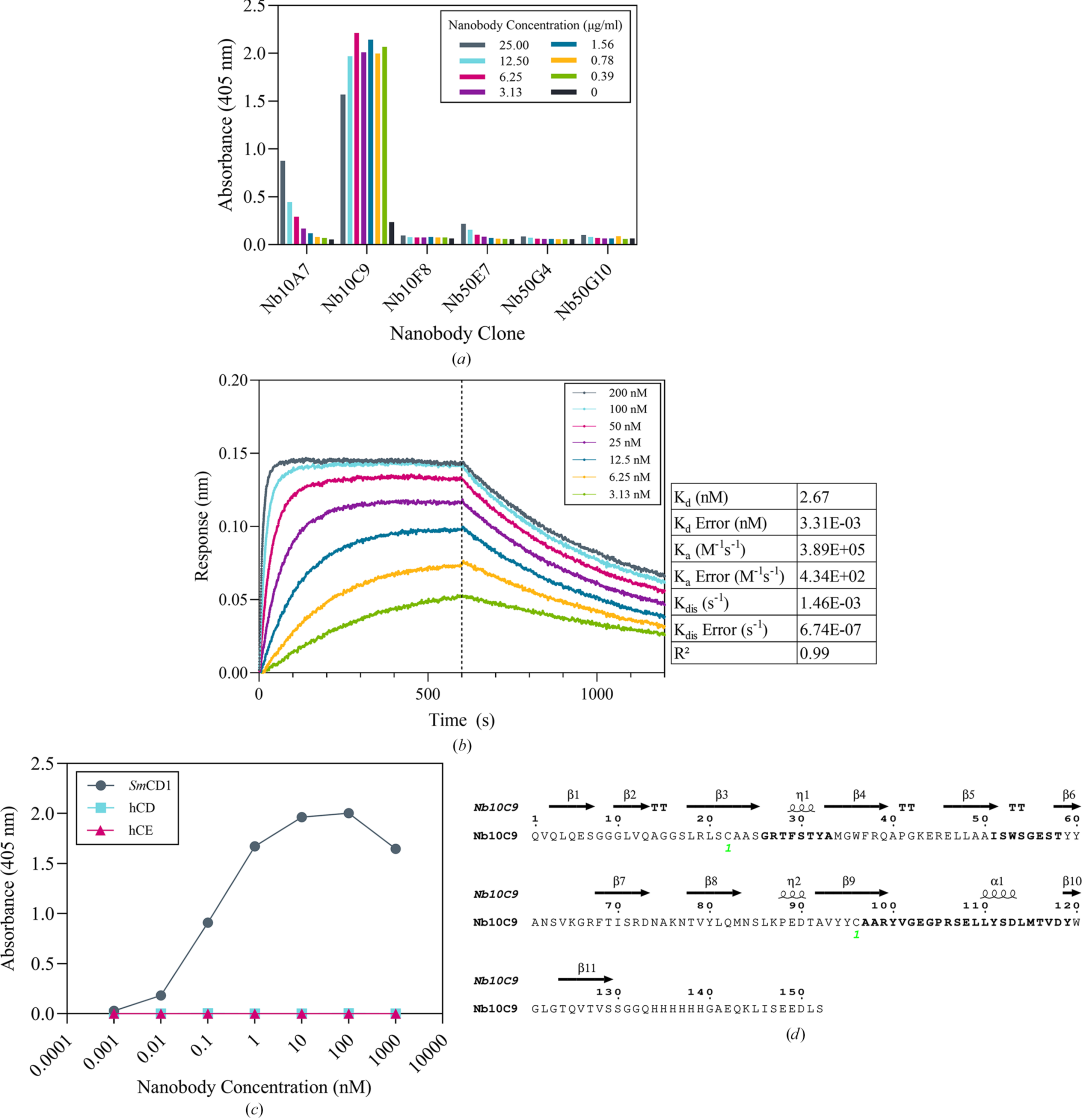
Characterization of nanobody binding. (*a*) Titration ELISAs using 50 n*MSm*CD1 immobilized in wells of a 96-well plate to assess the binding of purified nanobody, detected using anti-camelid VHH-HRP. Data shown represent single measurements from small-scale expressed material used for initial candidate prioritization. (*b*) Biolayer interferometry using streptavidin biosensors loaded with 50 n*M* biotinylated *Sm*CD1 binding to Nb10C9. Association occurred during the first 600 s; samples were then transferred to a buffer-containing well to measure dissociation for a further 600 s. Values shown in the table are weighted averages calculated from *n* = 3 technical replicates. Error values represent standard error of the mean. (*c*) Titration ELISA of Nb10C9 bound to *Sm*CD1, human cathepsin D (hCD) or human cathepsin E (hCE). (*d*) Amino-acid sequence of Nb10C9 with CDRs shown in bold (IMGT numbering; Giudicelli *et al.*, 2011 ▸), with secondary structures of Nb10C9 depicted along the top of the sequence. Green digits indicate disulfide bridges.

A total of 186 individual clones (93 from each round of panning) were randomly picked and assayed by ELISA, and the eight positive phage clones were sequenced. The results showed that all had unique CDR3 sequences and, of these, six clones were expressed on a small scale in *Escherichia coli* for preliminary binding studies. Titrations from 25 µg ml^−1^ identified a lead candidate, Nb10C9, which retained binding to *Sm*CD1 at the highest dilution (Fig. 2 ▸*a*). This nanobody was progressed to large-scale expression and successfully purified, yielding a solution at 22 mg l^−1^. Further titration ELISA and BLI allowed an estimation of affinity (Fig. 2 ▸*b*). Nb10C9 exhibited a strong interaction with *Sm*CD1, presenting a weighted average *K*_d_ of 2.67 n*M* (±3.31 × 10^−3^ n*M*), calculated from *n* = 3 technical replicates and fitted to a 1:1 binding model. ELISA showed that Nb10C9 did not bind to either human cathepsin D or E (Fig. 2 ▸*c*).

**Figure 3:**
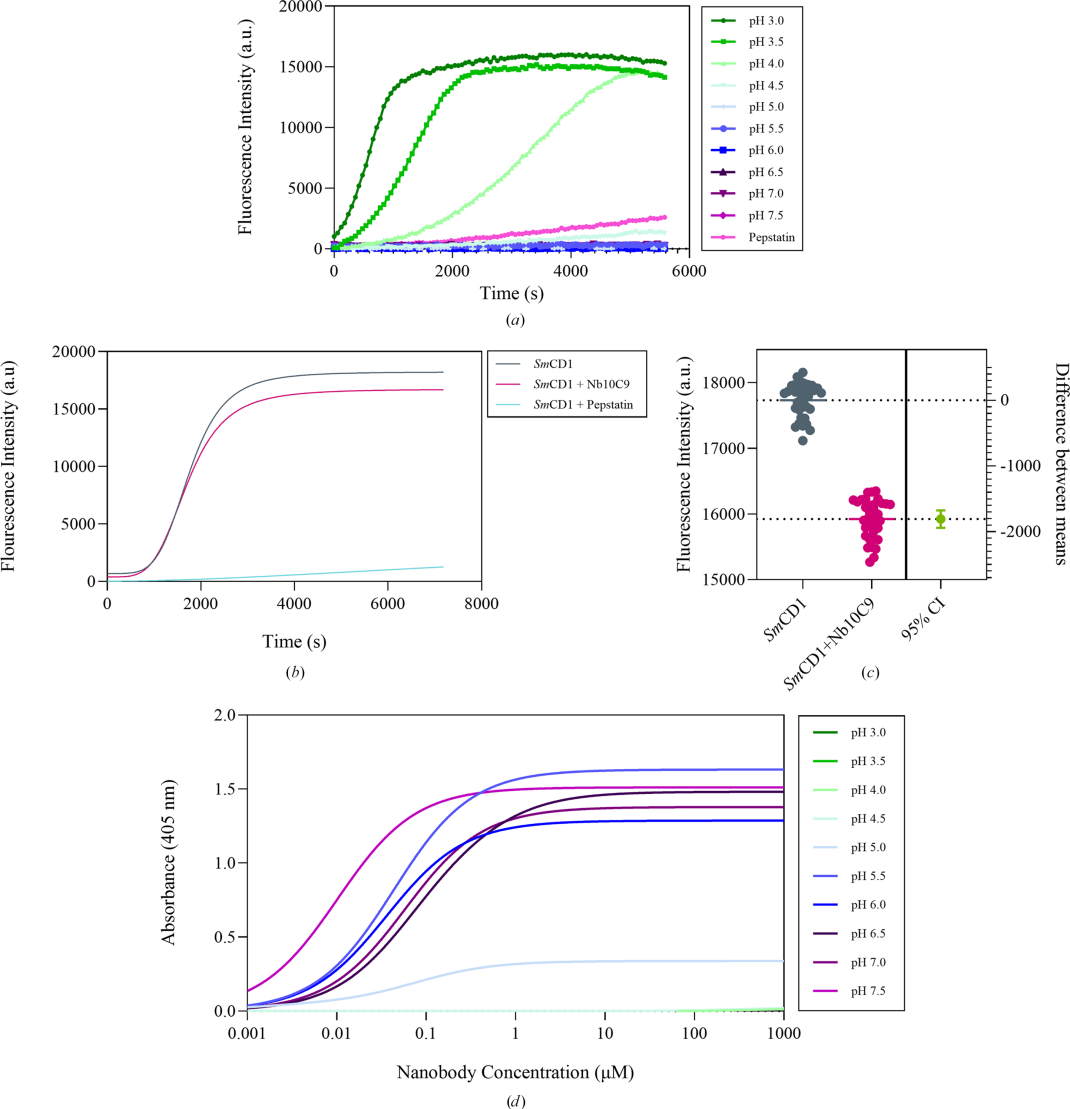
Activity of *Sm*CD1. (*a*) *Sm*CD1 activity measured over time under different pH conditions, recording the fluorescence of the fluorogenic substrate 7-methoxycoumarin-4-acetyl-GKPILFFRLK(DNP)-d-R-amide. (*b*) *Sm*CD1 activity at pH 3.5, incubated with 200 n*M* Nb10C9 or pepstatin. Data were background-subtracted and are displayed as the mean of *n* = 3 repeats. (*c*) Estimation plot of unpaired Student’s *t*-test for activity data of 50 n*MSm*CD1 or 50 n*MSm*CD1 + 200 n*M* Nb10C9 at the point of enzyme saturation, between 3000 and 4000 s. (*d*) Titration ELISA of Nb10C9 bound to 50 n*MSm*CD1 between pH 3.0 and pH 7.5. Data were background-subtracted and a line of best fit is displayed.

*Sm*CD1 is activated under acidic conditions, with optimal activity at pH 3–3.5, consistent with previous findings (Fig. 3 ▸*a*; Dunn, 2002 ▸; Araujo-Montoya *et al.*, 2020 ▸). Therefore, the pH-sensitivity of nanobody binding was assayed to determine whether the nanobody could affect enzyme activity. Kinetic assays suggested there may be a small inhibitory effect of Nb10C9 binding on *Sm*CD1 activity (Figs. 3 ▸*b* and 3 ▸*c*). However, this inhibition is limited by the pH-dependent binding of Nb10C9, which only shows titratable binding between pH 5.0 and 7.5 (Fig. 3 ▸*c*).

### 3.3. Structure of the *Sm*CD1–Nb10C9 complex

Nanobody Nb10C9 and *Sm*CD1 were mixed in a molar ratio of 1:1.2 (enzyme:nanobody), treated with EndoF1, co-purified (Supplementary Fig. S3) and crystallized at pH 6.0. The structure of the complex was solved using the experimentally determined *Sm*CD1 structure and a model-built structure of Nb10C9. Matthews coefficient analysis placed four copies of the *Sm*CD1–Nb10C9 ensemble within the asymmetric unit using *Phaser-MR* (Table 1 ▸). In the final model, four copies of *Sm*CD1 and two copies of Nb10C9 were observed within the asymmetric unit (Supplementary Fig. S4). Two distinct assemblies are noted, an *Sm*CD1 homodimer and an *Sm*CD1–Nb10C9 complex, that replicate across symmetry operator 00−100−1. Regular solvent channels throughout the crystalline layering prohibit sufficient space for additional copies of Nb10C9, with no evidence of obvious missing electron density.

For all copies of *Sm*CD1 (residues Val15–Pro385), connective density was observed for the entirety of the propeptide sequence and cleavage site (Ser49/Gly50) (Fig. 1 ▸*a* and Supplementary Fig. S1). Two of the four placed copies of *Sm*CD1 were in complex with two Nb10C9 nanobodies and showed complete connective backbone density over residues 15–385, including residues 46–53 of the propeptide sequence, a tract that was not resolved in the *Sm*CD1 structure (Fig. 1 ▸ and Supplementary Fig. S5). Otherwise, structural superimposition of *Sm*CD1 alone and with bound nanobodies showed that the two structures were identical, with an average r.m.s.d. of 0.28 Å between nanobody-bound chains of the complex and *Sm*CD1 alone (Supplementary Fig. S5). The unbound *Sm*CD1 molecules did not show connective density in this region, indicating that nanobody binding has stabilized the conformation of the propeptide towards the carboxyl end of the sequence.

**Figure 4:**
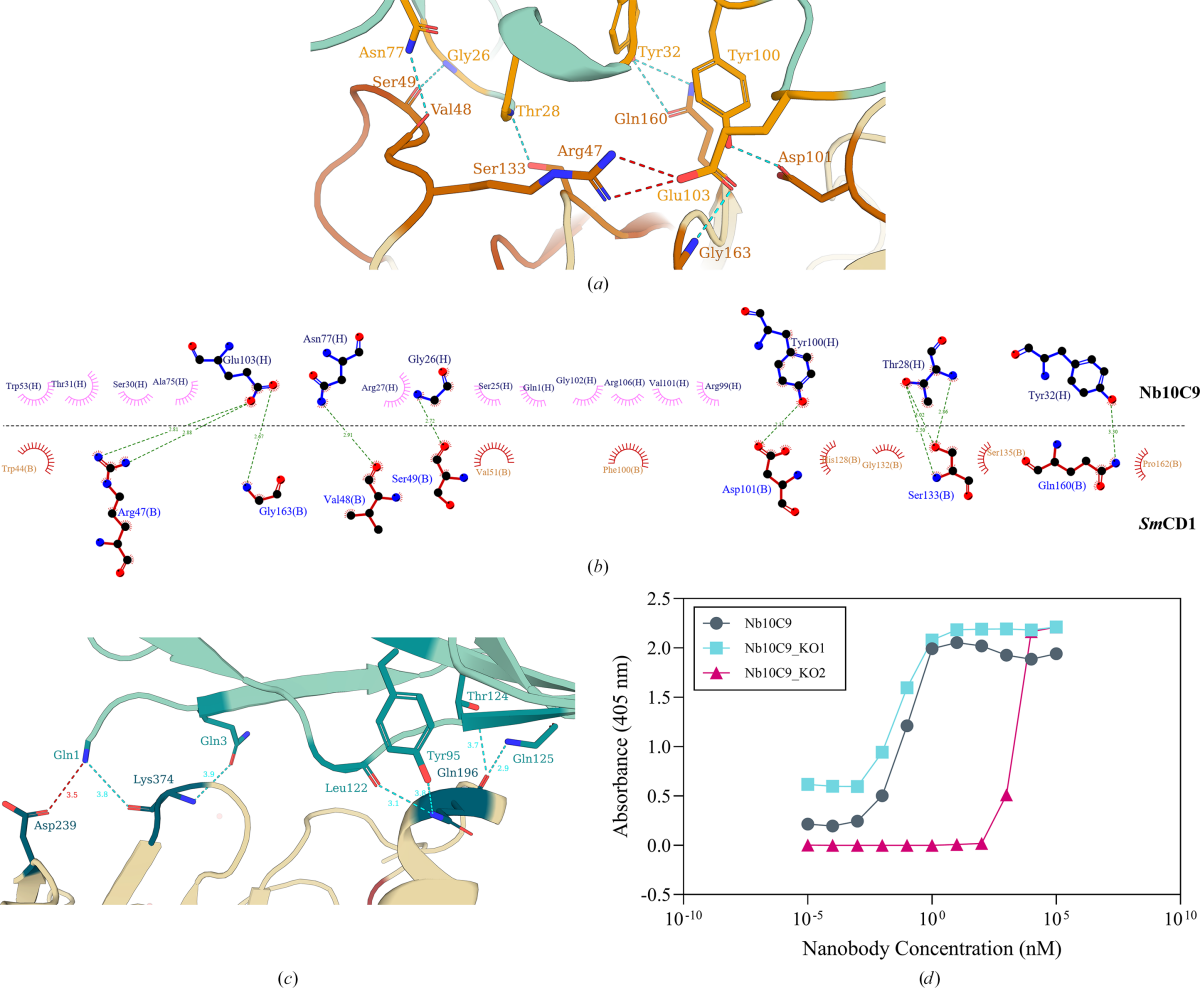
Molecular interactions between *Sm*CD1 and Nb10C9. (*a*) Molecular interactions between the backbone of *Sm*CD1 (light yellow) and Nb10C9 (turquoise), with the backbone of the propeptide sequence coloured orange. *PDBePISA*-identified interacting groups are shown in stick notation. Salt bridges are indicated by red dashed lines and hydrogen bonds by blue dashed lines. (*b*) *LigPlot*+ schematic (Laskowski & Swindells, 2011 ▸) of Nb10C9 (upper subpanel) and *Sm*CD1 (lower subpanel) residues participating in the CDR interface. Identified interactions are represented by dashed lines, whilst residues present at the interface are represented as surfaces. (*c*) Binding of *Sm*CD1 to Nb10C9 via the FR interface. Molecular interactions between the backbone of *Sm*CD1 (light yellow) and Nb10C9 (turquoise) are shown. Residues involved in the FR interface are coloured teal. *PDBePISA*-identified interacting groups are shown in stick notation. Salt bridges are indicated by red dashed lines and hydrogen bonds by blue dashed lines. Bond distances are represented in Å. (*d*) Titration ELISA of Nb10C9 mutants, Nb10C9_KO1 and Nb10C9_KO2, bound to 50 n*MSm*CD1.

Intermolecular interactions between Nb10C9 and *Sm*CD1 were identified using *PDBePISA* (Krissinel & Henrick, 2007 ▸). Nb10C9 recognizes residues positioned either side of the *Sm*CD1 active-site cleft, namely Arg47, Val48, Ser49, Asp101, Ser133, Gln160 and Gly163, described here as the ‘CDR interface’ (Figs. 4 ▸*a* and 4 ▸*b*). The critical interaction is a salt bridge between Glu103 of the Nb10C9 CDR3 and Arg47 of *Sm*CD1. Glu103 is stabilized by a hydrogen bond to the Gly163 amide, whilst Tyr100, also belonging to the Nb10C9 CDR3, hydrogen-bonds to Asp101 via side-chain contacts. Further hydrogen bonds are provided by the Nb10C9 CDR1. Backbone hydrogen bonds exist between residues Gly26 and Arg27 with Ser49 of *Sm*CD1 and between Thr28 and the Ser133 hydroxyl, and hydrogen bonds between the side chains of Tyr32 and Gln160. Finally, the carboxamide of framework-region Asn77 hydrogen-bonds to the carbonyl of Val48. A second binding site of Nb10C9 was also predicted by *PDBePISA* (Krissinel & Henrick, 2007 ▸) and by *PLIP* (Adasme *et al.*, 2021 ▸), suggesting an interaction between the framework regions of Nb10C9 and *Sm*CD1 (the ‘FR interface’). This secondary interaction primarily involves a salt bridge between Gln1 of Nb10C9 FR1 and Asp239 of *Sm*CD1 (Fig. 4 ▸*c*). Gln1 may be stabilized by a hydrogen-bond network involving Lys374 and Gln3, although the intermolecular distances captured within the crystal structure are strained at 3.75 and 3.87 Å, respectively. Gln196 also participates in the FR interface, forming hydrogen bonds to the phenolic hydroxyl of Tyr95 of FR3, adjacent to CDR3, as well as to Leu122, Thr124 and Gln125 of FR4. The secondary interaction between *Sm*CD1 and Nb10C9, via the FR interface, comprises only a handful of interactions which appear strained in the crystal structure and may be promoted by van der Waals forces.

Side-directed mutagenesis of Nb10C9 was carried out to test the importance of the interactions between Nb10C9 and *Sm*CD1 observed in the crystal structure for nanobody binding. Two Nb10C9 mutants were designed: Nb10C9_KO1, with the identified FR interface residues (Gln1, Gln3, Tyr95, Leu122, Thr124, Gln125) substituted by alanines, and Nb10C9_KO2, with alanine substitutions made for CDR interface residues (Gly26, Arg27, Thr28, Tyr32, Asn77, Tyr100, Gln103). Mutated nanobodies were expressed and purified and their binding activity was tested by ELISA compared with the parent nanobody (Fig. 4 ▸*d*). The results showed that Nb10C9_KO2 had reduced Nb10C9 binding, which was rationalized by the mutation of residues interacting with the propeptide, and confirmed the biological relevance of the CDR interaction. Interestingly, a low-affinity interaction persists at concentrations above 1 µ*M*. This could be attributed to the FR interaction, taking place with a low free energy of dissociation, since these residues are intact in the Nb10C9_KO2 mutant.

## 4. Discussion

We report the first crystal structure of schistosome cathepsin D1 as the inactive zymogen with the propeptide intact and covering the active site. A contiguous sequence for the propeptide was only observed in the structure of the enzyme in complex with a nanobody selected from a phage-display library following immunization of a llama with purified *Sm*CD1. This suggests that binding to the nanobody stabilizes the conformation of the propeptide. By analogy to human cathepsin D (Lee *et al.*, 1998 ▸), activation of the enzyme at acidic pH must involve a major conformational change such that following release of the propeptide the amino-terminal strand is rearranged to replace the β-strand of the propeptide in the six-stranded β-sheet between the two domains of the enzyme. In this way, the active site becomes accessible.

Comparison of the structures of *Sm*CD1 alone and in complex with Nb10C9 showed that the nanobody binds to the sequence Arg47–Ser49 immediately adjacent to the propeptide cleavage site between Ser49 and Gly50 (Brindley *et al.*, 2001 ▸). This sequence is identical in cathepsin D from *S. bovis*, and approximately 70% similar to the enzyme from *S. japonicum*, but differs from both human cathepsins D and E (Supplementary Fig. S2), consistent with the ELISA results (Fig. 2 ▸*c*). Therefore, Nb10C9 could be used as a schistosome-specific antibody for detecting *Sm*CD1 *in cellulo*, enabling localization studies within a native context.

Helminth infection, and the resulting schistosomiasis, remains difficult to treat and diagnose. Here, we have identified an epitope in *Sm*CD1 as a schistosome-specific biomarker and determined the structure of the *Sm*CD1 zymogen. This may guide future structure-based drug design for the development of novel therapeutics against schistosomiasis, a World Health Organization-recognized neglected tropical disease.

## 5. Related literature

The following references are cited in the supporting information for this article: Esparza *et al.* (2023 ▸), Robert & Gouet (2014 ▸) and Sievers & Higgins (2014 ▸, 2021 ▸).

## Supplementary Material

PDB reference: *Schistosoma mansoni*cathepsin D1, apo form, 9snt

PDB reference: bound to Nb10C9, 9rxi

Supplementary Methods and Supplementary Figures. DOI: 10.1107/S2059798326000422/ag5062sup1.pdf

## Figures and Tables

**Table 1:** X-ray crystallographic data-collection and refinement statistics Values in parentheses are for the outer shell.

	*Sm*CD1 (PDB entry 9snt)	*Sm*CD1–Nb10C9 (PDB entry 9rxi)
Data collection		
Space group	*P*6_2_	*P*12_1_1
*a*, *b*, *c* (Å)	136.62, 136.62, 51.80	70.14, 181.80, 78.85
α, β, γ (°)	90.00, 90.00, 120.00	90.00, 92.41, 90.00
Temperature (K)	293	293
Resolution range (Å)	44.72–2.20 (2.28–2.20)	90.90–2.59 (2.63–2.59)
*R*_merge_	0.292 (2.768)	0.671 (7.642)
*R*_meas_	0.314 (2.977)	0.699 (7.966)
*R*_p.i.m._	0.114 (1.080)	0.194 (2.202)
〈*I*/σ(*I*)〉	8.5 (1.1)	6.7 (1.0)
CC_1/2_	0.990 (0.311)	0.984 (0.262)
Completeness (%)	99.94 (100.00)	99.73 (100.00)
Multiplicity	7.4	12.1
Refinement		
Resolution range (Å)	44.72 (2.20)	90.90 (2.59)
No. of reflections	28306	86279
*R*_work_/*R*_free_	0.175/0.200	0.173/0.234
No. of atoms		
Protein	*A*, 2795	*A*, 5604; *B*, 5640: *C*, 5489; *D*, 5485; *E*, 1909; *H*, 1916
Ions/buffer	0	Ca, 2; Na, 2
Water	119	53
Total	2914	26100
Residual *B* factors (Å^2^)		
Protein	*A*, 40.14	*A*, 45.3; *B*, 46.1; *C*, 52.3; *D*, 63.4; *E*, 43.1; *H*, 40.6
Ligand/ion	0	Ca, 71.1, 51.7; Na, 79.7, 60.7
Water	42.93	34.8
R.m.s. deviations		
Bond lengths (Å)	0.008	0.0067
Bond angles (°)	0.95	1.68
Ramachandran plot		
Most favoured (%)	98.03	92.35
Clashscore	4.29	6

## Data Availability

The structural factors and atomic model coordinates for the *Sm*CD1 structure have been deposited in the wwPDB (Berman *et al.*, 2000 ▸) under accession code 9snt and for the *Sm*CD1–Nb10C9 co-crystal structure under accession code 9rxi.
