# Supplementary material for: Crystal structure of Schistosoma mansoni cathepsin D1 in complex with a nanobody reveals the conformation of the propeptide-bound state
Source: Acta Crystallogr D Struct Biol. 2026 Jan 28;82(Pt 2):140–50. doi: 10.1107/S2059798326000422 (PMC12865886; doi:10.1107/S2059798326000422)
Supplement: Supplementary file 1 [file d-82-00140-sup1.pdf]

STRUCTURAL
BIOLOGY

Volume 82 (2026)

Supporting information for article:

Crystal structure of *Schistosoma mansoni* cathepsin D1 in complex with a nanobody reveals the conformation of the propeptide-bound state

Kelly L. Parker, John D. Clarke, Xiaojiao Liu, Barbara F. Gomes, Lauren E.-A. Eyssen, Nicholas Furnham, Floriano Paes Silva-Jr and Raymond J. Owens

S1. Supplementary Methods

S1.1. Phage-Display Screening ELISAs

To evaluate success of the camelid immune response, a seroconversion ELISA was performed, using 10-fold dilutions of pre- and post-immune sera in wells coated overnight at 4ºC with 50 nM *Sm*CD1. 0.1 µg/mL Monorab™ Rabbit anti-Camelid VHH-HRP monoclonal antibody (A01861, GenScript) (diluted in 0.1 % (w/v) BSA-PBS) was used for detection of nanobody binders within the immune sera.

To confirm enrichment of binders for *Sm*CD1 through the biopanning process, a polyclonal ELISA was carried out. A 1:1 dilution of Library, Pan 1 and Pan 2 phage diluted in 2% (w/v) milk-PBS was used against wells coated overnight at 4 ºC with 50 nM *Sm*CD1. 'Enrichment' refers to an increase in absorbance signal as *Sm*CD1-specific phages are isolated through subsequent screening rounds.

Following colony picking, phage-containing supernatant from overnight TG1 *E. coli* cultures were harvested and used in an anti-M13 ELISA to analyse binding of individual nanobody clones. A 1:1 dilution of phage supernatant: 2 % (w/v) milk-PBS was added to ELISA wells coated overnight at 4 ºC with 50 nM *Sm*CD1.

For detection of bound-phage in both polyclonal and anti-M13 ELISAs, 0.25 µg/mL anti-M13-HRP mouse monoclonal antibody (11973-MM05T-H-SIB, Stratech) (diluted in 0.1 % BSA-PBS) was added. ABTS peroxidase substrate (LGC SeraCare) was added for antibody detection and incubated in the dark for 15 minutes. Absorbance was measured as microplate endpoint at 405 nm using a CLARIOStarPlus plate reader (BMG Labtech).

S1.2. pH Binding Studies

To further define the pH range of Nb10C9 binding, ELISA plates were coated with 50 nM *Sm*CD1 diluted in assay buffer (20 mM NaOAc, 150 mM NaCl) prepared at pH intervals of 0.5 in the range of pH 3.0-7.5. All washes between incubations, blocking solution (2% (w/v) milk in assay buffer) and Nb_10C9 were diluted in the corresponding assay buffer to retain each pH condition, akin to Esparza et al. (2023). Nb10C9 was titrated over a 10-fold dilution series from 1 µM - 0.001 µM. After incubation with Nb10C9, all wells were washed 3 times with 300 µL PBST at pH 7.5 to omit unbound nanobody and eliminate potential secondary effects on anti-VHH-HRP.

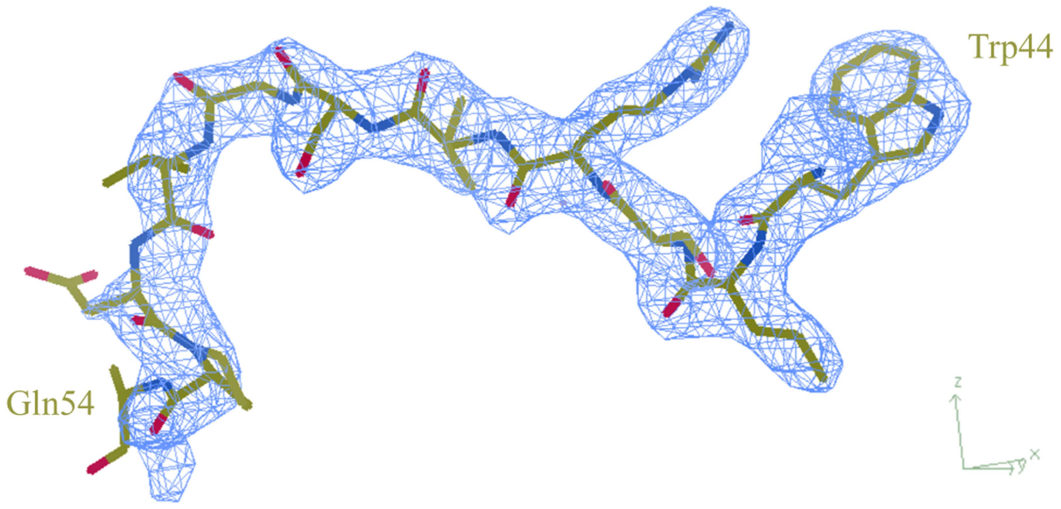

Figure S1 2mFo-Fc electron density map and atomic model illustrating N-terminal residues (Trp44 – Gln54) of the *Sm*CD1 propeptide. The fragment shown is data from the *Sm*CD1-Nb10C9 complex, since density for Ser45-Pro53 was not observed for the *Sm*CD1 apoenzyme. The atomic model is depicted as sticks and density map is shown as blue mesh contoured to a threshold of 1.00 σ.

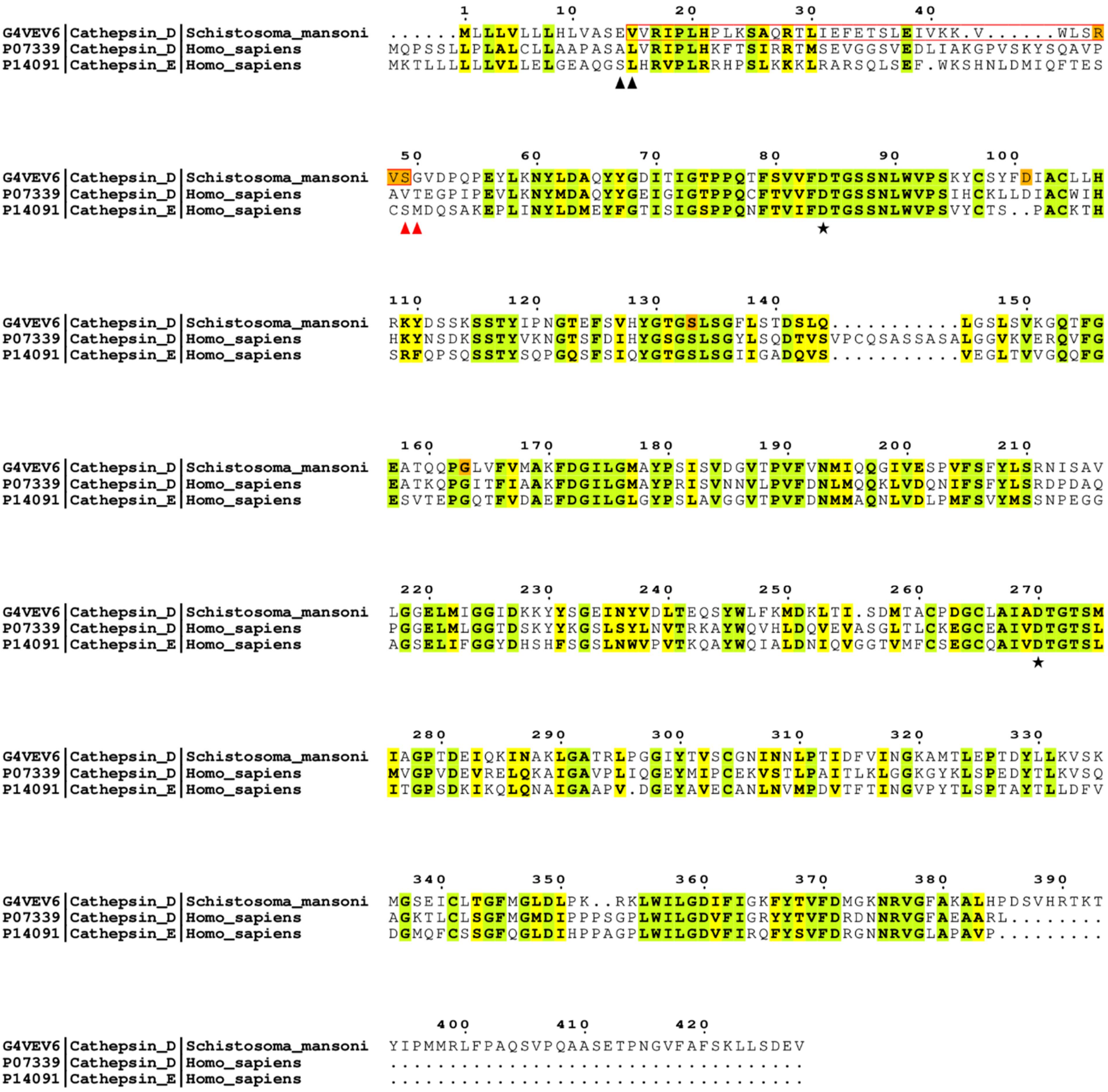

Figure S2 Multiple sequence alignment of *Schistosoma mansoni* Cathepsin D, human Cathepsin D and human Cathepsin E. Sequences are aligned using the Clustal Omega algorithm (Sievers & Higgins, 2014, 2021) and figure generated in ESPript (Robert & Gouet, 2014). Sequence similarity parameters are depicted based on a global score of 0.7, and conserved residues are in bold. Physiochemically equivalent residues are highlighted in yellow, while strictly conserved residues are highlighted in light green. The propeptide is outlined with a red box. The black arrows mark the signal sequence cleavage site, and the red arrows mark the propeptide cleavage site. The residues that interact with nanobody Nb10C9 are highlighted in orange and the two catalytic aspartate residues marked with black stars.

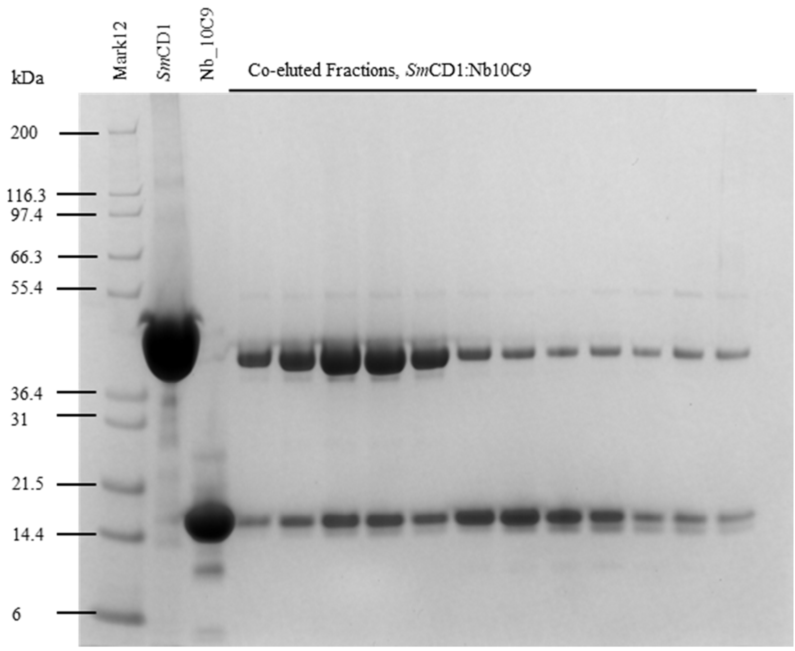

Figure S3 14-12% Bis-Tris SDS-PAGE Gel following co-purification of *Sm*CD1-Nb10C9 complex. The complex was purified on an ÄKTA Pure HPLC System with Superdex 200 10/300 GL Increase column. Fractions indicated were pooled and concentrated to 10 mg/mL.

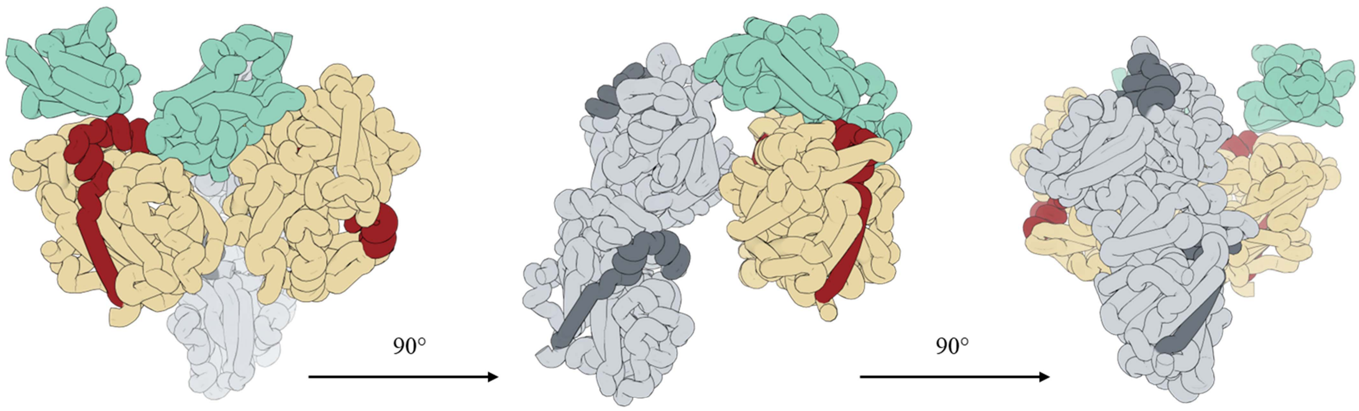

Figure S4 Overall crystal packing of *Sm*CD1-Nb10C9 complex, within the asymmetric unit. Chains of *Sm*CD1 monomer bound to Nb10C9 are depicted in light yellow, with the propeptide sequence highlighted in maroon. Unbound *Sm*CD1 chains are depicted in grayscale. Chains of Nb10C9 are shown in turquoise.

(a)

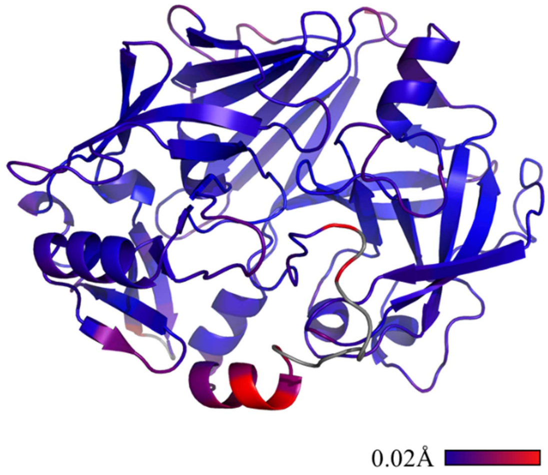

Chain	A	B	C	D	Apo
A	0	0.23	0.47	0.41	0.29
B	0.23	0	0.47	0.41	0.27
C	0.47	0.47	0	0.34	0.45
D	0.41	0.41	0.34	0	0.39
Apo	0.29	0.27	0.45	0.39	0

(b)

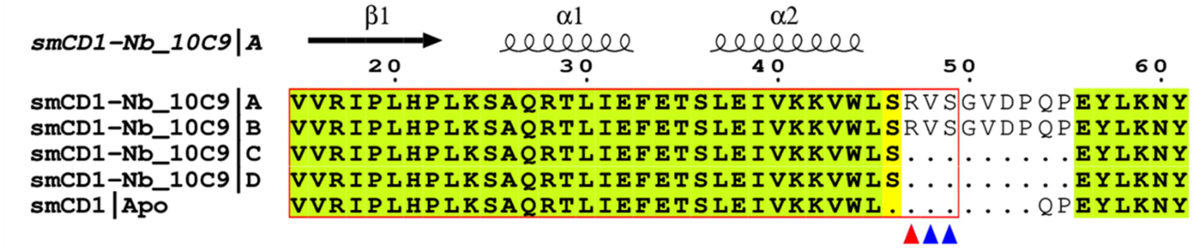

Figure S5 Alignment of *Sm*CD1, highlighting stabilisation of the active loop for chains bound to Nb10C9. (a) Alignment of *Sm*CD1 alone and in complex with Nb10C9 depicting the complex chain B, coloured by Root Mean Square Deviation (RMSD) between pairwise atoms of *Sm*CD1 apo-conformation. Atoms coloured in grey are lacking connective density in the *Sm*CD1 structure. Average RMSD values for pairwise alignments between all chains of *Sm*CD1-Nb10C9 and *Sm*CD1 structures are shown in the matrix ~~on~~ the right, with pairwise distances represented in Å. (b) Multiple sequence alignment of chains determined by XRD, from the *Sm*CD1 structure, as well as chains in the same ASU from the structure of *Sm*CD1-Nb10C9 complex. Residues 12-50 are displayed. Sequences are aligned using the Clustal Omega algorithm (Sievers & Higgins, 2014, 2021) and figure generated in ESPript (Robert & Gouet, 2014). The propeptide sequence (residues 15-54) is outlined with a red box. Binding sites for Nb10C9 are illustrated with triangles, red triangles represent salt bridges while blue triangles represent hydrogen bonds. Sequence similarity parameters are depicted based on a global score of 0.7, and conserved residues are bold. Physiochemically equivalent residues are highlighted in yellow, while strictly conserved residues are highlighted in light green. Secondary structures for *Sm*CD1 are depicted along the top of the alignment.
